# Pleural anthracosis presenting with massive effusion: a rare entity

**DOI:** 10.1002/rcr2.390

**Published:** 2018-11-28

**Authors:** Rayhan Amiseno, Andrea Yu‐Lin Ban, Noraidah Masir, Lizawati Rasul Hamidi, Mohamed Faisal Abdul Hamid

**Affiliations:** ^1^ Respiratory Department Universiti Kebangsaan Malaysia Medical Centre Kuala Lumpur Malaysia; ^2^ Pathology Department Universiti Kebangsaan Malaysia Medical Centre Kuala Lumpur Malaysia

**Keywords:** Anthracosis, carbon, granuloma, pneumoconiasis, tuberculosis

## Abstract

Pleural anthracosis is rare and, in most cases, is diagnosed incidentally or at autopsy. We report a 67‐year‐old man with pleural anthracosis. He was initially referred for possible tuberculous pleural effusion and had recurrent admissions for symptomatic pleural effusion, which increased with each subsequent episode. A thoracoscopic examination demonstrated diffuse hyperpigmentation in both parietal and visceral pleura. Parietal pleural biopsy indicated granuloma with foreign body giant cell. A contrast‐enhanced computed tomography (CECT) thorax showed focal plaques in parietal pleura with calcifications in the ipsilateral lung. Investigations for tuberculosis, fungal, and malignancy proved to be negative. With these results, a diagnosis of pleural anthracosis was made. This case highlights the unusual presentation of pleural anthracosis with pleural effusion.

## Introduction

Anthracosis is a form of pneumoconiosis caused by inhalation of carbon‐containing dust particles, which may affect various organs including the lungs. It varies from an asymptomatic individual to a more severe form of anthracofibrosis causing lung atelectasis. We describe a 65‐year‐old man with an unusual presentation of pleural anthracosis who presented with recurrent exudative pleural effusion. To our knowledge, this is the first reported case of biopsy‐proven pleural anthracosis presenting with massive pleural effusion.

## Case Report

A 65‐year‐old man with ischaemic stroke, hypertension, and end‐stage renal failure on regular haemodialysis presented with a two‐week history of shortness of breath and non‐productive cough. There was no history of fever, heart failure symptoms, or constitutional symptoms, and he had no limitations of his daily activities. He was compliant with fluid restrictions and achieved his target dry weight. He worked as a surveyor in a lead factory for 20 years and was a lifelong non‐smoker with no known exposure to asbestos.

Clinical examination demonstrated stony dullness and reduced breath sounds over the right lower hemithorax. He had an elevated white cell count of 20 × 10^9^/L and C‐reactive protein (CRP) of 10 mg/dL. Chest X‐ray (CXR) showed pleural effusion involving 25% of the right hemithorax. A diagnostic thoracentesis drained 1.5 L of exudative straw‐coloured fluid (pleural to serum protein ratio: 0.88, pleural to serum lactate dehydrogenase (LDH) ratio: 0.75). Gram‐positive cocci were detected in pleural fluid; however, the culture was negative. He was treated for a parapneumonic effusion with two weeks of antibiotics and was subsequently lost to follow‐up.

**Figure 1:**
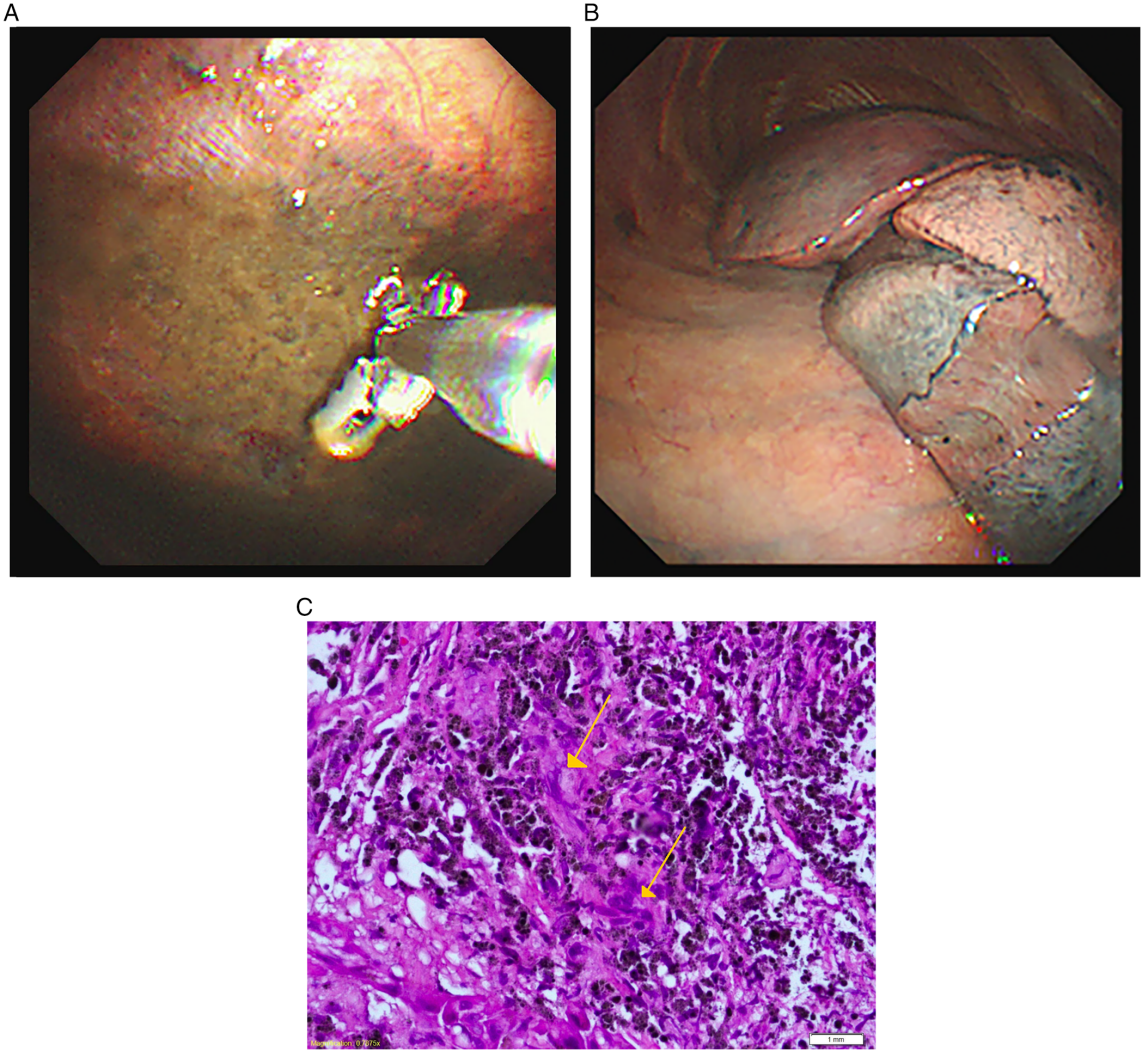
(A) Pleuroscopic examination demonstrated hyperpigmented lesions in the right parietal pleura whereby biopsy was taken (B) Hyperpigmented lesions in visceral pleura. (C) Histopathological examination under haematoxylin and eosin stain showed granuloma formation with foreign body‐type giant cells.

**Figure 2:**
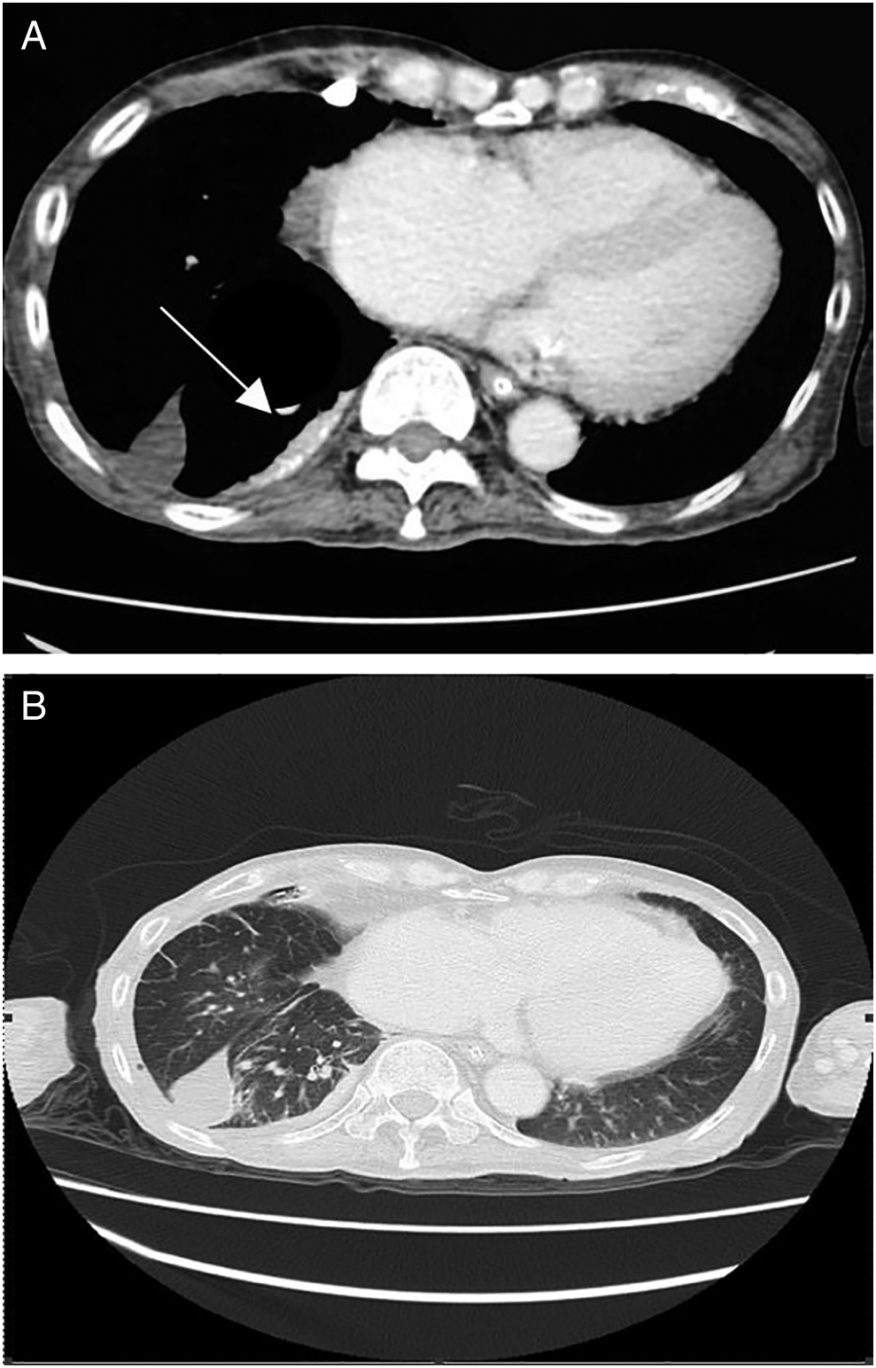
(A) Contrast‐enhanced computed tomography (CECT) thorax showed a 1.4 cm thick focal pleural plaque (white arrow) with calcification at the medio‐posterior aspect of right lower lobe. (B) Loculated fluid in the horizontal fissure and interlobular septal thickening.

He represented seven months later with a massive pleural effusion on the same side. A repeat thoracentesis was once again exudative (pleural to serum protein ratio: 0.68, pleural to serum LDH ratio: 0.85) with pleural pH of 8. Pleural fluid acid‐fast bacilli culture and sensitivity were negative. There were no malignant cells, and the cytospinned showed few lymphocytes admixed with neutrophils and macrophages. He was initially started on empirical broad‐spectrum antibiotics. We proceeded with a pleuroscopic examination (Fig. 1A and 1B), which demonstrated diffuse areas of hyperpigmentation at both parietal and visceral pleura. Histopathological examination of the parietal pleura (Fig. 1C) showed foreign body‐type granuloma with multinucleated giant cell. There was no evidence of fungal bodies and acid‐fast bacilli but evidence of malignancy. This was further supported by the findings from contrast‐enhanced computed tomography (CECT) of thorax (Fig. 2) performed two weeks later, which showed the presence of heterogeneously enhancing focal pleural plaque with foci calcification at the medio‐posterior aspect of the right lower lobe. Immunoglobulin levels and N‐terminal pro‐brain natriuretic peptide (NT‐proBNP) were not sent as they were not available in our centre.

He completed 14 days of antibiotics, and he was discharged well. A repeat chest radiograph a month later showed minimal pleural effusion. Bronchoscopy was not offered in view that repeated chest radiograph did not show any evidence of atelectasis as sequelae of anthracosis of the lung. He was given further follow‐up to monitor his progress.

## Discussion

The evaluation of pleural effusion in the medical wards starts with a history, clinical examination, and a chest radiograph. Thoracentesis will be the next step in deciding whether it is a transudate or an exudate using Light’s criteria 1.The pleural pH is usually helpful, but in our case, it may be inaccurate due to the sampling technique as our sample was not transported in an arterialized syringe and measured in blood gas analyser. A CECT thorax and thoracoscopic examination may be considered for further evaluation of the pleura.

Our patient underwent a diagnostic thoracentesis that demonstrated an exudative pleural effusion, which re‐accumulated despite antibiotics, and this warranted a thoracoscopic examination that indicated black carbon deposits on both pleura.

Anthracosis is a form of pneumoconiosis that is caused by the accumulation of carbon in the lungs due to repeated exposure to air pollution or inhalation of smoke or coal dust particles 2. Pulmonary anthracosis has been found in 3.4–21% of patients who underwent bronchoscopy for various reasons 3. Anthracosis has also been reported in the liver, spleen, and oesophagus 4. Pleural anthracosis, however, is a rare finding. Previous reported cases were associated with cocaine abuse and HIV infection 5.

Our patient had pigment deposition on direct visualization suggestive of carbon deposits, which we believe to be anthracosis. This was proven by biopsy. Computed tomography (CT) scan findings of anthracosis in most cases has been reported as calcified mediastinal or hilar lymphadenopathy, followed by bronchial thickening and stenosis. 3 Our patient had heterogenous‐enhancing focal pleural plaque with foci calcification, which further supports the diagnosis of pleural anthracosis.

We hypothesize that the likely cause of the pleural effusion is pleural anthracosis due to the presence of black carbon pigments on the calcified pleural plaques on CT thorax and the biopsy report. Due to the lack of data from the literature about pleural anthracosis, we opted for a conservative approach with close monitoring. In conclusion, anthracosis is a rare condition, and the diagnosis requires a thorough history, specific imaging, and endoscopic examinations as well as exclusion of other diagnosis. The treatment for anthracosis is not well established and mainly conservative and treats the underlying condition.

### Disclosure Statement

Appropriate written informed consent was obtained for publication of this case report and accompanying images.
